# Clinical Factors Associated with Diagnostic Delay in Axial Spondyloarthritis in Saudi Arabia: A Retrospective Cohort Study

**DOI:** 10.3390/diagnostics16193246

**Published:** 2026-10-08

**Authors:** Mohamed Bedaiwi, Aos Aboabat, Ibrahim Almaghlouth, Eman Alqurtas, Norah Alshafi

**Affiliations:** Rheumatology Unit, Department of Medicine, College of Medicine, King Saud University, Riyadh 11461, Saudi Arabia

**Keywords:** axial spondyloarthritis, diagnostic delay, fibromyalgia, clinical diagnosis, Saudi Arabia, Arabian Gulf

## Abstract

**Background/Objectives**: Diagnostic delay remains a major challenge in axial spondyloarthritis (axSpA), yet factors associated with delay in Saudi Arabia and the Arabian Gulf remain poorly characterized. We examined clinical factors associated with diagnostic delay in a single-center Saudi cohort. **Methods**: This retrospective cohort included 160 of 220 screened registry patients with rheumatologist-confirmed axSpA who fulfilled ASAS classification criteria. Diagnostic delay was measured in whole years from documented symptom onset to diagnosis. Twenty-four patients had fibromyalgia documented before axSpA diagnosis. The primary negative binomial model included sex, axSpA subtype, HLA-B27 status, documented prediagnosis fibromyalgia, and symptom-onset age; logistic regression evaluated delay > 5 years. Standardized predictions estimated absolute differences, and a 2-year landmark analysis reduced differential opportunity for fibromyalgia ascertainment. **Results**: Median delay was 3 years (IQR 2–6), and 41 patients (25.6%) had delay >5 years. Documented prediagnosis fibromyalgia was associated with longer expected delay (ratio of expected diagnostic delays, 1.59; 95% CI 1.24–2.04; *p* < 0.001), corresponding to an adjusted difference of 2.33 years (bootstrap 95% CI 0.87–4.13), and with greater odds of delay >5 years (OR 5.56; 95% CI 2.08–14.85). Older symptom-onset age was also associated with longer delay. In the landmark analysis (*n* = 107), fibromyalgia documented by 2 years remained associated with longer subsequent delay (ratio of expected diagnostic delays, 1.51; 95% CI 1.05–2.19; *p* = 0.027). **Conclusions**: Documented prediagnosis fibromyalgia and older symptom-onset age were associated with longer diagnostic delay. The fixed-window landmark analysis reduced concern about differential ascertainment opportunity but remained subject to selection and reverse causation and did not establish causality. Persistent features suggestive of inflammatory axial disease in patients with fibromyalgia warrant selective diagnostic reconsideration.

## 1. Introduction

Axial spondyloarthritis (axSpA) is a chronic inflammatory musculoskeletal disease that encompasses radiographic and nonradiographic forms and typically begins in early adulthood [1]. Diagnosis is often delayed for several years because chronic back pain is common, inflammatory features may be incompletely recognized, objective findings may be absent early in the disease course, and referral pathways may be fragmented. A meta-analysis estimated a mean diagnostic delay of approximately 6.7 years, whereas a later systematic review found median estimates commonly ranging from 2 to 6 years, with substantial variation across populations, settings, and calendar periods [2,3]. International data further show that diagnostic delay is influenced by geographic, sociodemographic, clinical, and health-system factors [4,5].

Delayed diagnosis can prolong uncertainty, repeated consultations, inappropriate investigations, work disruption, and cumulative symptom burden while postponing access to disease-specific management [6,7,8]. However, reported correlates of delay are inconsistent. Age at symptom onset, sex, HLA-B27 status, disease phenotype, previous alternative diagnoses, healthcare-provider sequence, and social vulnerability have all been implicated, but the direction and magnitude of these associations vary across cohorts [9,10]. Region-specific analyses are therefore important because findings from other populations or referral systems may not transfer directly to Saudi or Gulf practice.

The Saudi and regional evidence base is growing but remains limited and fragmented. Recent Saudi studies have described remission, imaging features, treatment patterns, and other aspects of axSpA care [11,12]. Only a small number have directly examined diagnostic delay: one included a heterogeneous spondyloarthritis population, and a recent MRI-confirmed cohort compared delay by HLA-B27 status [12,13]. These studies were informative but were largely descriptive, heterogeneous, or subgroup-focused. None modeled patient-level diagnostic delay in an axSpA-specific multivariable negative binomial analysis, translated relative associations into adjusted differences in years, or internally validated the estimates with bootstrap resampling.

This gap is important because diagnostic pathways, case mix, and the availability and interpretation of objective findings may differ across regions. Saudi imaging data also show substantial diagnostic delay in both HLA-B27-positive and HLA-B27-negative subgroups [12]. Region-specific empirical data are therefore needed to identify patients at risk of delayed recognition and to inform future pathway evaluation.

Fibromyalgia is particularly relevant because it can coexist with axSpA and complicate symptom attribution. Widespread pain, fatigue, tenderness, sleep disturbance, and functional impairment may overlap with symptoms reported during evaluation for inflammatory disease [14]. A Colombian study found that a prior fibromyalgia diagnosis was associated with longer axSpA diagnostic delay [9]. This does not imply that fibromyalgia is an incorrect diagnosis; rather, a valid pain diagnosis may coexist with unrecognized inflammatory disease and potentially contribute to diagnostic anchoring. Because fibromyalgia is diagnosed more frequently in women and sex-associated differences in axSpA recognition have been reported [15,16], we also examined a sex-by-fibromyalgia interaction as an explicitly exploratory supplementary analysis.

We therefore used an updated, independently verified Saudi institutional cohort to identify clinical factors associated with diagnostic delay. The primary objective was to model delay using variables fixed or established during the diagnostic period and to translate relative associations into adjusted differences in years. Secondary objectives were to evaluate delay exceeding 5 years and assess the robustness of the principal findings. A sex-by-fibromyalgia interaction was examined exploratorily and is reported in the Supplementary Materials.

## 2. Materials and Methods

### 2.1. Study Design and Setting

We conducted a single-center retrospective cohort study in the rheumatology service of a tertiary academic hospital in Riyadh, Saudi Arabia. Data were obtained from an ongoing institutional axSpA registry linked to electronic medical records. The registry was originally approved by the King Saud University Institutional Review Board on 3 May 2020 (protocol E-20-4626; Ref. No. 20/0223/IRB) and has remained under active IRB renewal, most recently Renewal No. 5 approved on 15 June 2026 (Ref. No. 26/0464/IRB). Patients entered the analytic cohort through baseline assessments conducted between January 2021 and April 2025, with clinical follow-up available through April 2026. The present diagnostic-delay study was a separate retrospective secondary analysis and received a study-specific exempt determination on 19 July 2026 (IRB No. R26-IRB-566; Ref. No. 26/0570/IRB), with a waiver of informed consent. Historical records predating registry entry were reviewed to reconstruct the chronology of axSpA-attributable symptom onset, fibromyalgia documentation, and axSpA diagnosis. Reporting followed the Strengthening the Reporting of Observational Studies in Epidemiology (STROBE) statement.

**Figure 1:**
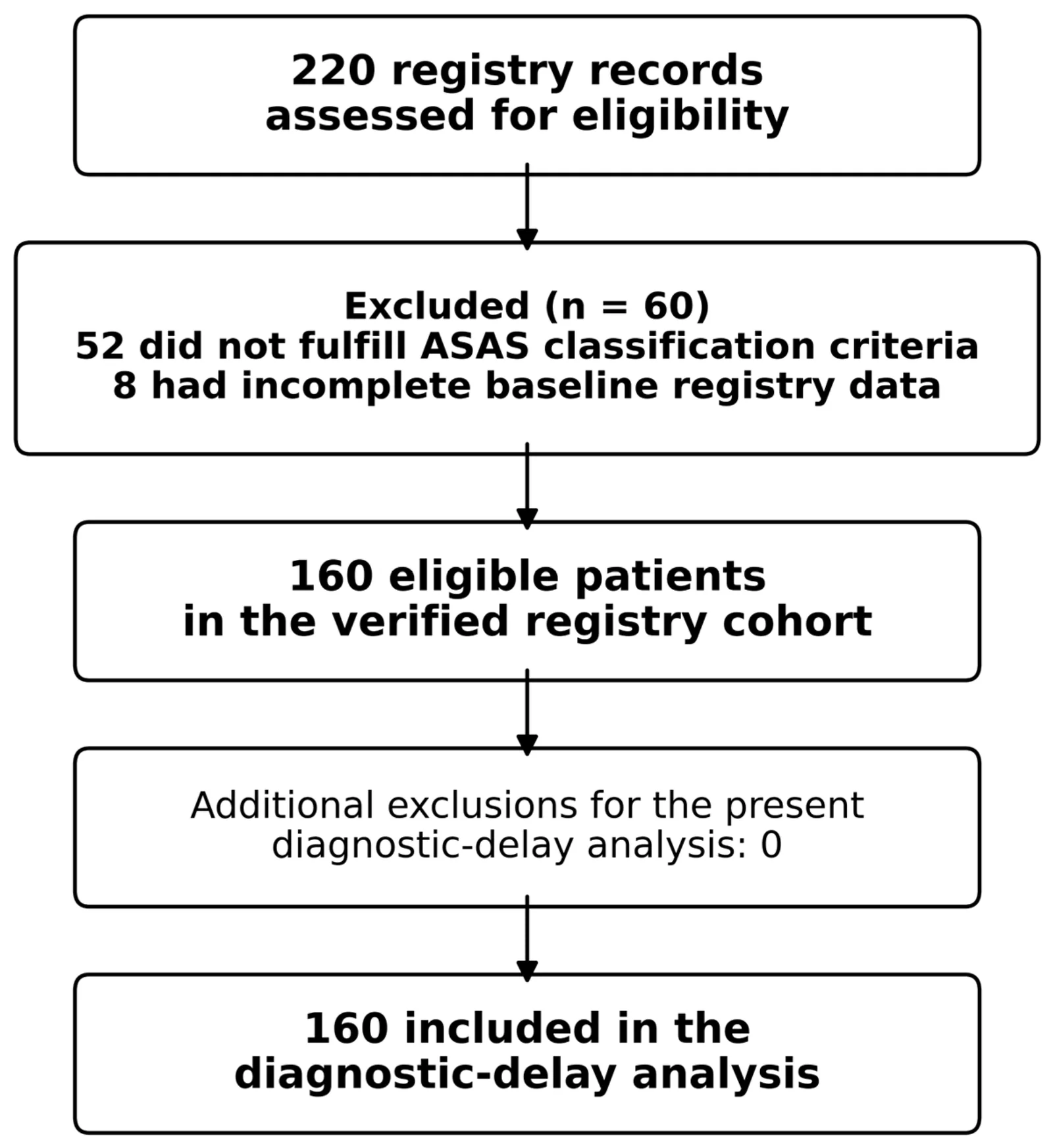
Participant flow from registry screening to the diagnostic-delay analytic cohort. ASAS, Assessment of SpondyloArthritis international Society.

All patients recorded in the institutional axSpA registry during the eligibility period were screened. Of 220 records, 52 did not fulfill the ASAS classification criteria for axSpA and 8 had incomplete baseline registry data, leaving 160 patients in the verified analytic cohort. The final de-identified analytic dataset was verified and locked on 22 July 2026, after the study-specific IRB determination; no patients or data were added thereafter. All 160 patients had complete recorded ages at symptom onset and axSpA diagnosis, as well as complete data for the five primary covariates selected before model fitting; therefore, no additional patients were excluded from the present diagnostic-delay analysis (Figure 1).

A 2026 report based on the same 160-patient registry cohort primarily evaluated sex differences in disease-activity measures and their association with concomitant fibromyalgia [17]. That report described diagnostic delay and included an exploratory adjusted comparison by sex, but it did not examine documented prediagnosis fibromyalgia as a correlate of diagnostic delay, model delay using negative binomial regression, evaluate prolonged delay, generate adjusted delay predictions, or perform a fixed-window landmark analysis. The present study therefore addresses a distinct primary question using the same verified 160-patient registry cohort.

A separate 2026 Clinical Rheumatology publication included 148 patients, all of whom constitute a nested subset of the present 160-patient cohort [18]. Both studies arose from the same ongoing institutional registry; however, the Clinical Rheumatology analysis applied additional eligibility requirements, including sufficient clinical information and paired baseline and approximately 12-month ASDAS-CRP measurements, and used a treatment-episode anchor for longitudinal outcome assessment. Its primary aims were to describe clinical phenotype, comorbidity burden, cumulative advanced-therapy exposure, and 12-month disease-activity outcomes. Several demographic and clinical variables therefore overlap with the present study, and mean diagnostic delay was previously reported descriptively as 4.3 ± 3.3 years. However, diagnostic delay was not analyzed as an outcome in that publication, fibromyalgia timing relative to axSpA diagnosis was not reconstructed for that purpose, and no multivariable analysis of diagnostic-delay correlates was performed. Thus, although the source population and several descriptive variables overlap, the analytic eligibility criteria, study anchors, primary questions, and outcome analyses are distinct.

### 2.2. Participants

The analytic cohort comprised the 160 eligible adults identified through the registry screening process described above. All had rheumatologist-confirmed axSpA, fulfilled the Assessment of SpondyloArthritis international Society (ASAS) classification criteria [19], and had complete data for diagnostic delay and the five primary covariates. Fulfillment of the ASAS classification criteria was verified retrospectively at registry entry using the available historical clinical, laboratory, and imaging documentation, irrespective of the calendar year of the original axSpA diagnosis. For patients with older diagnoses, eligibility therefore did not rely solely on the historical diagnostic label. Imaging information used for this verification was derived from available radiology reports and treating-rheumatologist documentation; original images were not centrally re-read. No values were imputed, and no further exclusions were applied. Radiographic and nonradiographic axSpA were defined according to the treating rheumatologist’s diagnosis and available imaging documentation. The source registry excluded patients with concomitant rheumatoid arthritis, systemic lupus erythematosus, or another systemic inflammatory rheumatic disease from the eligible axSpA cohort. Psoriasis and inflammatory bowel disease were permitted as recognized axSpA-associated manifestations. No formal sample-size calculation was performed because all eligible patients in the verified cohort were included.

### 2.3. Data Collection and Definitions

Clinical data were abstracted from the longitudinal electronic medical record using a structured chart-abstraction form with predefined operational definitions. Because identifying the first axSpA-attributable symptom required clinical interpretation of the historical record, this key variable was independently abstracted by two trained reviewers, with the study fibromyalgia classification masked during this assessment. Disagreements regarding symptom attribution or timing were resolved by joint review of the source documentation and consensus. Formal inter-reviewer agreement statistics were not calculated. Diagnostic delay was calculated as age at rheumatologist-confirmed axSpA diagnosis minus age at the first symptom documented in the rheumatology history as attributable to axSpA. Symptom-onset age was derived from the chronology documented in rheumatology histories and cross-checked against available earlier records. Exact dates were not consistently available; therefore, delay was recorded in whole years and may be subject to recall, rounding, and retrospective classification error.

Prediagnosis fibromyalgia was classified as present only when an explicit diagnosis of fibromyalgia by a rheumatologist was documented before the axSpA diagnosis. A nonspecific mention of widespread pain or tenderness, or an isolated diagnostic code without a documented clinical diagnosis, was insufficient. Repeated rheumatologist documentation and/or criteria-compatible clinical features served as corroborating evidence, and all 24 patients classified as having documented prediagnosis fibromyalgia met this documentation standard. The specific formal classification criteria used in routine care were not consistently recorded. Diagnosis timing was verified for each exposed patient. For the landmark sensitivity analysis, reviewers additionally abstracted the interval in months between the first documented fibromyalgia diagnosis and the first axSpA-attributable symptom for all 24 exposed patients. The remaining 136 patients were classified as having no documented prediagnosis fibromyalgia; this category does not imply the absence of fibromyalgia diagnosed later. Fibromyalgia documented after axSpA diagnosis was not systematically ascertained and was outside the scope of this analysis. Exact onset dates of fibromyalgia symptoms were not consistently available. This exposure definition establishes the temporal order of documented diagnoses but does not demonstrate that fibromyalgia symptoms preceded axial symptoms or caused diagnostic delay.

The five primary covariates were sex, radiographic versus nonradiographic axSpA, HLA-B27 status, documented prediagnosis fibromyalgia, and age at symptom onset. They were selected before model fitting because they were fixed characteristics or were established during the diagnostic period and were clinically relevant to axSpA recognition. Current smoking, body mass index (BMI), and hypertension were measured at the later index assessment and could have arisen or changed after the diagnostic interval; they were therefore excluded from the primary adjustment set and evaluated only in an extended sensitivity model. Covariates were not selected on the basis of univariable *p*-values.

### 2.4. Outcomes

The primary outcome was diagnostic delay in years. A secondary binary outcome defined prolonged delay as more than 5 years; this threshold was selected before fitting the logistic model because it represented a clinically meaningful upper segment of the cohort while retaining 41 events. Disease-activity measures obtained at the later index assessment were treated as descriptive characteristics only and were moved to the Supplementary Materials because they could not explain the preceding diagnostic interval. The sex-by-fibromyalgia interaction was designated as exploratory before fitting the interaction model.

### 2.5. Statistical Analysis

Continuous variables are summarized as median and interquartile range (IQR), and categorical variables as number and percentage. Descriptive between-group comparisons used the Mann–Whitney U test or Fisher’s exact test, as appropriate. Diagnostic delay was right-skewed, integer-valued, and overdispersed relative to a Poisson distribution. We therefore fitted multivariable negative binomial regression with a log link. Because the outcome represents elapsed duration rather than an incidence count, exponentiated coefficients are described as ratios of expected diagnostic delays, with 95% confidence intervals (CIs) and two-sided *p*-values. Model choice was assessed using the Pearson dispersion statistic, the negative binomial overdispersion parameter, and Akaike information criterion (AIC). Variance inflation factors were used to assess collinearity.

To express the fibromyalgia association in years, standardized adjusted predictions were generated by setting the documented prediagnosis fibromyalgia indicator first to absent and then to present for every participant while retaining the remaining covariates at their observed values. Predicted delays were averaged across the cohort, and the difference was interpreted as a model-based adjusted absolute contrast. Percentile CIs were obtained from 2000 nonparametric bootstrap resamples using the fixed seed 20260725; 1995 primary-model resamples converged.

The same five variables were included in logistic regression for delay exceeding 5 years. Because the prolonged-delay model included 41 events across five primary predictors, Firth penalized-likelihood logistic regression was also fitted as a small-sample sensitivity analysis. For the exploratory supplementary analysis, a sex-by-fibromyalgia product term was added to the primary negative binomial model while retaining hierarchical main effects. Nested models were compared using a likelihood-ratio test, and standardized predicted delays were estimated for the four sex/prediagnosis fibromyalgia groups. Bootstrap CIs were based on 1997 converged resamples. Because only eight men had documented prediagnosis fibromyalgia, the interaction analysis was considered hypothesis-generating.

Sensitivity analyses included an extended eight-variable negative binomial model additionally adjusting for current smoking, BMI, and hypertension; an analogous eight-variable logistic model; median quantile regression; and refitting the primary model after excluding eight observations with delay greater than 10 years. The functional form of symptom-onset age was assessed using a three-knot restricted cubic spline with knots at the 10th, 50th, and 90th percentiles. Analyses were performed using Python 3.12, NumPy 2.3.5, SciPy 1.17.0, and statsmodels 0.14.6. A two-sided *p*-value below 0.05 was considered statistically significant, with emphasis placed on effect estimates and CIs.

To reduce differential opportunity for fibromyalgia ascertainment, we performed a targeted 2-year landmark sensitivity analysis. The landmark was chosen as a pragmatic fixed early window that gave all included patients the same 24-month opportunity for fibromyalgia documentation while retaining an adequate analytic sample and number of exposed patients. Eligibility required axSpA diagnosis more than 2 years after documented symptom onset, so all included patients remained undiagnosed throughout the initial 24 months. This restriction necessarily selects patients with longer diagnostic intervals; accordingly, the landmark analysis was treated as a sensitivity analysis rather than an unbiased estimate for the full cohort. Fibromyalgia was classified as present at the landmark if it had been documented before symptom onset or within the first 24 months after symptom onset; patients without such documentation by 24 months formed the comparison group. The outcome was the remaining diagnostic delay from the 2-year landmark to axSpA diagnosis. The negative binomial model used the same adjustment variables as the primary model (sex, axSpA subtype, HLA-B27 status, and age at symptom onset, with landmark fibromyalgia status replacing the primary fibromyalgia indicator). Standardized predictions were bootstrapped with 2000 resamples (fixed seed 20260807). An analogous adjusted logistic model examined eventual delay exceeding 5 years. A formal time-dependent survival analysis was not performed because exact dates of axSpA-attributable symptom onset and axSpA diagnosis were not consistently available across the cohort; the primary outcome was reconstructed from recorded ages and therefore had one-year granularity. Although the timing of first fibromyalgia documentation relative to symptom onset was abstracted in months for the 24 exposed patients, the outcome timing was not sufficiently precise to support a reliable time-dependent analysis.

### 2.6. Use of Generative Artificial Intelligence-Assisted Tools

During manuscript preparation and revision, the authors used ChatGPT-5.6 (OpenAI; accessed July–September 2026) to assist with language editing, manuscript organization, document formatting, and drafting statistical analysis code. The authors independently reran all analyses using the verified dataset; reviewed and corrected all AI-assisted output; verified the numerical results and references; and take full responsibility for the accuracy, integrity, and originality of the submitted work. No artificial intelligence-assisted technology was listed as an author or cited as a source of scientific evidence.

## 3. Results

### 3.1. Cohort and Diagnostic-Delay Distribution

The cohort included 160 patients: 104 men (65.0%) and 56 women (35.0%); 90 (56.2%) had radiographic axSpA, 70 (43.8%) had nonradiographic axSpA, and 100 (62.5%) were HLA-B27-positive. Twenty-four patients (15.0%) had documented prediagnosis fibromyalgia; the remaining 136 had no documented prediagnosis fibromyalgia. Median diagnostic delay was 3 years (IQR 2–6), the mean was 4.31 years (standard deviation 3.02), and the range was 1–17 years. No patient had a recorded diagnostic delay of zero years. Forty-one patients (25.6%) experienced delay exceeding 5 years (Supplementary Table S1). The median calendar year of axSpA diagnosis was 2018 (IQR 2015–2021; range 1992–2025).

**Figure 2:**
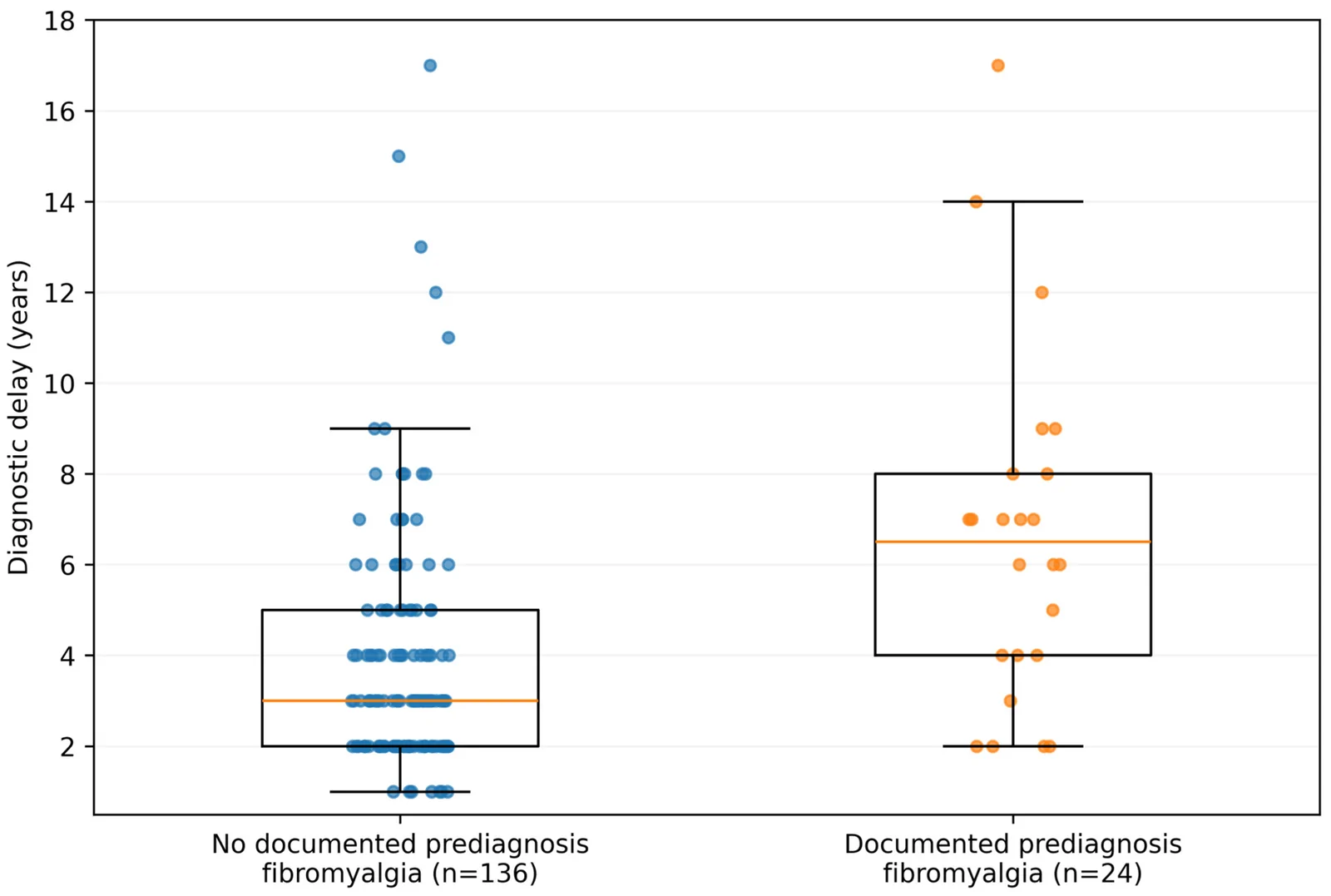
Diagnostic delay by documented prediagnosis fibromyalgia status. Box plots show medians and interquartile ranges. Individual patient points are displayed with slight horizontal jitter to reduce overlap caused by whole-year recording of diagnostic delay. Fibromyalgia diagnosed after axSpA was not systematically ascertained.

Patients with documented prediagnosis fibromyalgia were more likely to be women (66.7% versus 29.4%), had a longer median diagnostic delay (6.5 versus 3 years), and more frequently experienced delay exceeding 5 years (62.5% versus 19.1%) (Table 1). Disease-activity measures obtained at the later index assessment are presented descriptively in Supplementary Table S5 and are not interpreted as consequences of diagnostic delay. Figure 2 shows the distribution of diagnostic delay by documented prediagnosis fibromyalgia status.

### 3.2. Primary Multivariable Analysis

The Poisson model showed overdispersion (Pearson dispersion 1.75). The negative binomial overdispersion parameter was 0.121 (*p* < 0.001), and the negative binomial model had a lower AIC than the Poisson model (727.49 versus 747.82). Variance inflation factors for all five primary covariates were below 1.3. Detailed model diagnostics and bootstrap validation are provided in Supplementary Table S4.

In the primary negative binomial model, documented prediagnosis fibromyalgia was associated with a 59% longer expected diagnostic delay (ratio of expected diagnostic delays, 1.59; 95% CI 1.24–2.04; *p* < 0.001) (Table 2). In the primary linear age specification, each 5-year increase in symptom-onset age was also associated with longer delay (ratio of expected diagnostic delays, 1.10; 95% CI 1.04–1.17; *p* = 0.002). This per-5-year estimate should be interpreted as a parsimonious average association across the observed age range rather than as a constant effect at every age. Sex, axSpA subtype, and HLA-B27 status were not independently associated with delay.

Standardized predicted mean delay was 3.945 years when documented prediagnosis fibromyalgia was set to absent and 6.271 years when it was set to present, yielding an adjusted difference of 2.33 years (bootstrap 95% CI 0.87–4.13). The bootstrap median ratio of expected diagnostic delays for fibromyalgia was 1.58, with a percentile 95% CI of 1.21–2.08.

### 3.3. Prolonged Diagnostic Delay

In logistic regression, documented prediagnosis fibromyalgia was associated with more than fivefold greater odds of diagnostic delay exceeding 5 years (OR 5.56; 95% CI 2.08–14.85; *p* < 0.001). A Firth penalized-likelihood sensitivity model yielded a concordant estimate (OR 5.08; 95% profile-likelihood CI 2.01–13.53; *p* < 0.001). In the primary linear age specification, each 5-year increase in symptom-onset age was also associated with prolonged delay (OR 1.37; 95% CI 1.06–1.78; *p* = 0.016). Sex, axSpA subtype, and HLA-B27 status were not independently associated with the binary outcome.

### 3.4. Exploratory Supplementary Analysis

The exploratory sex-by-fibromyalgia interaction term was 1.94 (95% CI 1.14–3.30; *p* = 0.015) and remained above the null in bootstrap analysis. However, only eight men had documented prediagnosis fibromyalgia, and subgroup estimates were imprecise. Detailed estimates and standardized predictions are reported in Supplementary Table S6 and Supplementary Figure S1; these findings are hypothesis-generating.

### 3.5. Sensitivity Analyses

The fibromyalgia association remained stable across alternative specifications (Table 3). The extended eight-variable negative binomial model yielded a ratio of expected diagnostic delays of 1.55 (95% CI 1.21–2.00) (Supplementary Table S2). Median quantile regression estimated an adjusted median difference of 2.80 years (95% CI 1.75–3.85). Excluding delays greater than 10 years yielded a ratio of expected diagnostic delays of 1.49 (95% CI 1.21–1.85), and the eight-variable logistic model yielded an OR of 5.42 (95% CI 1.97–14.93) for delay exceeding 5 years (Supplementary Table S3). The restricted cubic spline sensitivity analysis suggested modest departure from strict log-linearity for symptom-onset age (likelihood-ratio *p* = 0.043; Supplementary Figure S2), while the fibromyalgia estimate remained materially unchanged (ratio of expected diagnostic delays, 1.56; 95% CI 1.22–2.00; Supplementary Table S4). The linear per-5-year age estimate is therefore interpreted as a parsimonious average association across the observed age range.

Among the 24 patients with documented prediagnosis fibromyalgia, three (12.5%) had fibromyalgia documented before the first recorded axSpA-attributable symptom, 19 (79.2%) during the first 24 months after symptom onset, and two (8.3%) more than 24 months after symptom onset but before axSpA diagnosis. Thus, 22 of 24 patients (91.7%) had fibromyalgia documented by 2 years after axSpA symptom onset. Four of these 22 patients received their axSpA diagnosis within the first 2 years and were therefore ineligible for the landmark cohort, leaving 18 patients with fibromyalgia documented by the landmark; the two patients whose fibromyalgia was first documented after 24 months were classified as unexposed at the landmark.

The targeted 2-year landmark analysis included 107 patients who remained undiagnosed beyond 2 years after symptom onset. In this cohort, median total diagnostic delay was 7.0 years (IQR 5.3–8.0) among patients with fibromyalgia documented by the landmark versus 4.0 years (IQR 3.0–6.0) among those without such documentation. After adjustment for sex, axSpA subtype, HLA-B27 status, and symptom-onset age, fibromyalgia documented by the landmark remained associated with longer subsequent diagnostic delay (ratio of expected diagnostic delays, 1.51; 95% CI 1.05–2.19; *p* = 0.027). Standardized expected remaining delay was 4.88 versus 3.22 years, an adjusted difference of 1.66 years (bootstrap 95% CI 0.23–3.74; 1999/2000 resamples converged). The adjusted odds of eventual delay exceeding 5 years were also higher (OR 4.66; 95% CI 1.35–16.04; *p* = 0.015) (Supplementary Table S7).

## 4. Discussion

In this retrospective analytical cohort study, documented prediagnosis fibromyalgia and older symptom-onset age remained associated with longer diagnostic delay after multivariable adjustment. The fibromyalgia association was consistent across the negative binomial model, a clinically interpretable threshold of more than 5 years, median regression, exclusion of extreme delays, an extended covariate model, and bootstrap validation. The relative estimate corresponded to approximately 2.3 additional adjusted years, while the binary analysis showed more than fivefold greater odds of prolonged delay. Convergence across these analyses reduces the likelihood that the finding was driven solely by skewness, extreme observations, or a single modeling choice.

The study also addresses a regional evidence gap. Saudi axSpA research has expanded, but most available studies have focused on remission, imaging, phenotype, or treatment outcomes [11,12,17,18]. The few Saudi reports addressing diagnostic delay were descriptive, heterogeneous, subgroup-focused, or treated delay as a secondary cohort characteristic [12,13,18]. To our knowledge, this is the first Saudi axSpA-specific study to evaluate multiple patient-level correlates of diagnostic delay using an overdispersed negative binomial model, report an adjusted absolute contrast in years, analyze a prolonged-delay threshold, and internally validate the estimates.

Region-specific data are important because the diagnostic context varies across healthcare systems and patient populations. Access to MRI, specialty of first contact, recognition of inflammatory back pain, and interpretation of HLA-B27 can influence referral and diagnosis. Saudi imaging data have shown substantial diagnostic delay across HLA-B27 subgroups [12]. The present findings therefore provide region-specific evidence that may inform future education and pathway evaluation rather than assuming that international predictors apply unchanged.

The cohort’s median diagnostic delay of 3 years and mean delay of 4.31 years were lower than the overall International Map of Axial Spondyloarthritis (IMAS) estimates (median 4.0 years; mean 7.4 years) but closely matched the Asian IMAS mean of 4.2 years [4]. They were also similar to the 2.9-year median reported in the international ASAS-COMOSPA study [5] and fell within the 2–6-year range of medians summarized in a systematic review [3]. The mean delay was also consistent with the 4.3 ± 3.3 years previously reported descriptively in the nested 148-patient cohort from our center [18]; the present study extends that descriptive observation by examining patient-level correlates and the timing of documented prediagnosis fibromyalgia. Direct international comparisons remain limited by differences in calendar period, case mix, symptom-onset ascertainment, and healthcare systems. Importantly, 25.6% of patients still experienced delays longer than 5 years, showing that an acceptable overall median can conceal a clinically important high-delay subgroup.

The association with documented prediagnosis fibromyalgia provides the clearest point of international concordance. A Colombian axSpA cohort similarly found that a prior fibromyalgia diagnosis was associated with longer diagnostic delay [9]. The present study extends that observation by verifying temporal documentation, quantifying the association in both relative and absolute terms, and specifically addressing differential exposure opportunity. Twenty-two of the 24 prediagnosis fibromyalgia diagnoses (91.7%) were documented by 2 years after axSpA symptom onset, and the association remained directionally similar when all included patients were given the same 24-month ascertainment window in the landmark analysis (adjusted ratio of expected diagnostic delays, 1.51). This makes it less likely that the primary association is explained solely by patients with longer delays having more time in which a fibromyalgia diagnosis could be recorded. Fibromyalgia can coexist with inflammatory disease and should not automatically be regarded as a misdiagnosis. Once widespread pain, fatigue, tenderness, and sleep disturbance are attributed to fibromyalgia, subsequent axial symptoms may be interpreted within that framework and inflammatory features may be revisited less readily. The reverse pathway remains possible, however: a prolonged undifferentiated pain journey may itself increase the probability that fibromyalgia is recognized. The landmark analysis reduces, but cannot eliminate, this possibility and does not establish causality.

The higher BASDAI, BASFI, and ASDAS-CRP values observed descriptively among patients with documented prediagnosis fibromyalgia should also be interpreted in the context of previous evidence showing that concomitant fibromyalgia can increase patient-reported and composite disease-activity measures and may affect apparent treatment response, often without comparable differences in objective inflammatory markers [20,21,22]. These later disease-activity findings remain descriptive and are not interpreted as consequences of diagnostic delay.

Older symptom-onset age was also associated with longer delay, but the direction differed from several large international datasets. IMAS associated younger symptom onset with longer delay [4], whereas ASAS-COMOSPA and a United States cohort found that older onset was associated with a greater probability of early or shorter diagnosis [5,10]. In our setting, later onset may reduce clinical suspicion for axSpA because degenerative explanations may appear more plausible; however, this interpretation is hypothesis-generating and requires replication. A restricted cubic spline sensitivity analysis suggested modest departure from strict log-linearity, and the adjusted-prediction curve is shown in Supplementary Figure S2. Accordingly, the per-5-year estimate from the primary linear model should be regarded as a parsimonious average association across the observed age range rather than as a constant effect at every age. Differences in referral systems, diagnostic awareness, case mix, and calendar period may contribute to the direction observed in this cohort.

The exploratory supplementary interaction suggested that the association between documented prediagnosis fibromyalgia and delay may differ by sex, but the small number of exposed men produced imprecise estimates. The pattern is compatible with the literature describing sex-associated differences in axSpA presentation and recognition [15,16], but it should not be considered definitive or used to support sex-specific diagnostic rules. Replication in a larger multicenter cohort is required.

Female sex was not independently associated with delay in the primary model. This contrasts with IMAS and ASAS-COMOSPA, in which female sex was associated with longer delay or lower odds of early diagnosis [4,5], although the broader literature has not shown a fully consistent sex effect across settings [3,15,16]. The exploratory interaction raises the possibility that sex-related differences may be concentrated among patients with documented prediagnosis fibromyalgia rather than operating as a uniform independent effect, but the small exposed male subgroup precludes firm conclusions. Radiographic subtype and HLA-B27 status were likewise not independently associated with delay. Final subtype and HLA-B27 results may become available late in the diagnostic process and therefore may not reflect the information available to initial providers. Regional imaging studies have also shown substantial delay across HLA-B27 subgroups [12]. More direct pathway variables, including specialty of first contact, number of providers, timing of MRI, and prior alternative diagnoses, may be more informative but were not consistently available in the present registry.

The clinical implication is not to disregard fibromyalgia or to investigate all patients with widespread pain indiscriminately. Rather, a documented fibromyalgia diagnosis should not close diagnostic reasoning when persistent features remain suggestive of inflammatory axial disease. In selected patients, focused reassessment or repeat rheumatology evaluation may be reasonable, consistent with the literature describing diagnostic and primary-care barriers in axSpA [23,24,25,26,27]. However, the present study did not evaluate a specific reassessment or referral pathway and cannot determine whether such an approach would shorten diagnostic delay. Any structured pathway therefore requires prospective evaluation before implementation.

Strengths include the use of a complete, independently verified dataset; a primary adjustment set restricted to variables fixed or established during the diagnostic period; a negative binomial model appropriate for overdispersed integer-valued delay; standardized predictions expressed in years; a clinically interpretable prolonged-delay model; bootstrap validation; and a fixed-window landmark sensitivity analysis specifically designed to reduce differential opportunity for fibromyalgia ascertainment. The relationships to earlier Saudi studies and publications from the same registry were explicitly distinguished and disclosed.

Several limitations require emphasis. The single-center tertiary-care setting may enrich for complex or longstanding disease and limits generalizability. Diagnostic delay was reconstructed retrospectively from recorded ages rather than uniformly available exact dates and therefore has one-year granularity and potential recall and classification error. Although the first axSpA-attributable symptom was independently abstracted by two reviewers using predefined operational definitions, with the study fibromyalgia classification masked during this assessment and disagreements resolved by consensus, retrospective symptom attribution remains dependent on the quality and detail of the original clinical documentation; formal inter-reviewer agreement was not assessed. Earlier nonspecific or widespread pain may not always be distinguishable retrospectively from manifestations subsequently attributed to axSpA. Prediagnosis fibromyalgia required an explicit rheumatologist diagnosis with corroborating documentation, but the specific formal classification criteria used in routine care were not consistently recorded, so some exposure misclassification remains possible. Because fibromyalgia could be documented at any time before axSpA diagnosis, the primary exposure definition is vulnerable to differential exposure opportunity: patients with longer diagnostic intervals have more time in which a fibromyalgia diagnosis can be recorded. The 2-year landmark sensitivity analysis reduced this concern by applying the same 24-month ascertainment window to all included patients and produced a concordant association. However, the landmark cohort was necessarily restricted to patients who remained undiagnosed at 2 years, creating a selected subgroup with systematically longer diagnostic intervals. The landmark analysis was also smaller (18 exposed patients); depended on retrospectively reconstructed symptom chronology; and cannot eliminate reverse causation, selection related to remaining undiagnosed, residual timing error, or unmeasured confounding.

Fibromyalgia after axSpA diagnosis was not systematically ascertained; therefore, the 136-patient comparison group represents no documented prediagnosis fibromyalgia rather than absence of fibromyalgia at any time. The cohort included axSpA diagnoses spanning 1992–2025, during which MRI availability, recognition of nonradiographic axSpA, referral pathways, and clinical documentation practices evolved. Calendar period was not included in the primary regression models, and residual confounding by secular changes in diagnostic practice cannot be excluded. Other potentially important confounders, including socioeconomic status, health literacy, provider sequence, family history, detailed inflammatory back-pain components, prediagnostic imaging, and dates of extra-musculoskeletal manifestations, were not available in analysis-ready form. The interaction analysis was exploratory and included only eight exposed men. Finally, these observational associations cannot establish that changing a diagnostic label or referral pathway would reduce delay.

Future multicenter Saudi and Gulf studies should reconstruct the diagnostic pathway prospectively or through detailed chart review. Larger cohorts would primarily improve the precision of effect estimates and statistical power, particularly for subgroup, interaction, and landmark analyses with relatively few exposed patients; estimated magnitudes may change with larger samples even though the principal fibromyalgia association was consistent across the present sensitivity analyses. High-value variables include exact symptom and diagnosis dates, fibromyalgia onset and diagnosis dates, the number and specialties of healthcare professionals seen, prior mechanical or degenerative diagnoses, imaging chronology, HLA-B27 testing date, referral source, geographic access, and socioeconomic measures. Such studies would test the generalizability of the present findings, quantify health-system contributors, and evaluate whether structured reassessment among selected patients with fibromyalgia shortens time to rheumatology review without promoting indiscriminate testing or overdiagnosis.

## 5. Conclusions

Documented prediagnosis fibromyalgia and older symptom-onset age remained associated with longer diagnostic delay after multivariable adjustment in this Saudi axSpA cohort. Documented prediagnosis fibromyalgia corresponded to approximately 2.3 additional adjusted years and more than fivefold greater odds of delay exceeding 5 years. The fixed-window 2-year landmark analysis yielded a concordant association with subsequent delay, reducing concern about differential ascertainment opportunity while remaining vulnerable to selection and reverse causation. These findings support maintaining diagnostic openness when patients with fibromyalgia have persistent features suggestive of inflammatory axial disease. Prospective evaluation is required before any specific reassessment pathway can be considered validated.

## Figures and Tables

**Table 1:** Characteristics by documented prediagnosis fibromyalgia status.

Characteristic	Overall (*N* = 160)	No Documented Prediagnosis Fibromyalgia (*n* = 136)	Documented Prediagnosis Fibromyalgia (*n* = 24)	*p*-Value
Age, years	40 (34–48)	39 (32–47)	44 (37.5–53.3)	0.055
Female sex	56 (35.0%)	40 (29.4%)	16 (66.7%)	<0.001
Age at symptom onset, years	29 (22–35)	29 (22–35)	32.5 (23.3–35.3)	0.377
Age at axSpA diagnosis, years	33 (26–40)	32 (25–38.3)	37.5 (31.3–46.3)	0.034
Diagnostic delay, years	3 (2–6)	3 (2–5)	6.5 (4–8)	<0.001
Diagnostic delay > 5 years	41 (25.6%)	26 (19.1%)	15 (62.5%)	<0.001
Nonradiographic axSpA	70 (43.8%)	56 (41.2%)	14 (58.3%)	0.126
HLA-B27-positive	100 (62.5%)	86 (63.2%)	14 (58.3%)	0.654
Current smoking	36 (22.5%)	27 (19.9%)	9 (37.5%)	0.067
Body mass index, kg/m^2^	27.4 (23.8–30.8)	27.1 (23.7–30.0)	30.1 (26.9–32.4)	0.011
Hypertension	23 (14.4%)	18 (13.2%)	5 (20.8%)	0.346

Values are median (IQR) or n (%). *p*-values reflect descriptive, unadjusted comparisons. axSpA, axial spondyloarthritis; HLA-B27, human leukocyte antigen B27.

**Table 2:** Primary models of diagnostic delay and delay exceeding 5 years.

Predictor	Adjusted Expected-Delay Ratio or OR	95% CI	*p*-Value
Panel A: primary negative binomial model			
Female sex	1.06	0.86–1.31	0.581
Nonradiographic versus radiographic axSpA	1.16	0.94–1.42	0.171
HLA-B27-positive	1.03	0.83–1.27	0.789
Documented prediagnosis fibromyalgia	1.59	1.24–2.04	<0.001
Age at symptom onset, per 5 years	1.10	1.04–1.17	0.002
Panel B: logistic model for delay > 5 years			
Female sex	1.96	0.85–4.51	0.116
Nonradiographic versus radiographic axSpA	1.36	0.56–3.30	0.494
HLA-B27-positive	1.28	0.53–3.10	0.585
Documented prediagnosis fibromyalgia	5.56	2.08–14.85	<0.001
Age at symptom onset, per 5 years	1.37	1.06–1.78	0.016

Panel A reports ratios of expected diagnostic delays; Panel B reports odds ratios (ORs). Both primary models included sex, axSpA subtype, HLA-B27 status, documented prediagnosis fibromyalgia, and age at symptom onset. These variables were fixed or established during the diagnostic period and were selected before model fitting.

**Table 3:** Sensitivity analyses of the documented prediagnosis fibromyalgia association.

Analysis	Effect Measure	Estimate	95% CI	*p*-Value
Primary five-variable negative binomial model	Expected-delay ratio	1.59	1.24–2.04	<0.001
Extended eight-variable negative binomial model	Expected-delay ratio	1.55	1.21–2.00	<0.001
Median quantile regression	Adjusted median difference	2.80 years	1.75–3.85	<0.001
Primary model excluding delay > 10 years	Expected-delay ratio	1.49	1.21–1.85	<0.001
Bootstrap primary-model estimate	Expected-delay ratio	1.58	1.21–2.08	-
Standardized adjusted absolute contrast	Difference	2.33 years	0.87–4.13	-
Extended eight-variable logistic model for delay >5 years	OR	5.42	1.97–14.93	0.001
Firth penalized logistic model for delay >5 years	OR	5.08	2.01–13.53	<0.001
Two-year landmark negative binomial model (*n* = 107)	Expected-delay ratio	1.51	1.05–2.19	0.027
Landmark standardized remaining-delay contrast	Difference	1.66 years	0.23–3.74	-

Note: The quantile-regression estimate is an adjusted conditional median difference, whereas the standardized negative binomial contrast is a marginal difference in model-predicted mean diagnostic delay; these estimands are not directly comparable. The Firth confidence interval was obtained from the penalized profile likelihood. The extended models additionally adjusted for current smoking, BMI, and hypertension measured at the later index assessment. The primary bootstrap used 2000 resamples; 1995 fits converged. The 2-year landmark analysis was restricted to patients with diagnostic delay > 2 years, classified fibromyalgia using only documentation available by 24 months after symptom onset, and modeled the remaining delay after the landmark; its standardized contrast used 2000 bootstrap resamples (1999 converged; seed 20260807). Expected-delay ratio denotes the exponentiated negative binomial coefficient, interpreted as the ratio of expected diagnostic delays.

## Data Availability

The datasets generated and/or analyzed during the current study are not publicly available because public release could compromise participant confidentiality and is restricted under institutional and ethics requirements. A de-identified minimum dataset may be available from the corresponding author on reasonable request and subject to approval by King Saud University and the responsible ethics committee.

## Supplementary Materials

The following supporting information can be downloaded at https://www.mdpi.com/article/10.3390/diagnostics16193246/s1, Supplementary Table S1: Characteristics by diagnostic-delay threshold; Supplementary Table S2: Extended eight-variable negative binomial model; Supplementary Table S3: Extended eight-variable logistic model for delay exceeding 5 years; Supplementary Table S4: Model diagnostics, bootstrap valida-tion, and additional sensitivity checks; Supplementary Table S5: Later index-assessment disease-activity measures by documented prediagnosis fibromyalgia status; Supplementary Table S6: Exploratory sex-by-documented-prediagnosis-fibromyalgia analyses; Supplementary Table S7: Two-year landmark sensitivity analysis for fibromyalgia ascertainment; Supplementary Figure S1: Adjusted diagnostic delay by sex and documented prediagnosis fibromyalgia status; Supplementary Figure S2: Adjusted relationship between age at axSpA symptom on-set and predicted diagnostic delay.

## Abbreviations

AIC: Akaike information criterion; ASAS: Assessment of SpondyloArthritis international Society; ASDAS-CRP: Ankylosing Spondylitis Disease Activity Score using C-reactive protein; axSpA: axial spondyloarthritis; BASDAI: Bath Ankylosing Spondylitis Disease Activity Index; BASFI: Bath Ankylosing Spondylitis Functional Index; BMI: body mass index; CI: confidence interval; HLA-B27: human leukocyte antigen B27; IQR: interquartile range; OR: odds ratio.
