# Supplementary material for: Clinical Factors Associated with Diagnostic Delay in Axial Spondyloarthritis in Saudi Arabia: A Retrospective Cohort Study
Source: Diagnostics (Basel). 2026 Oct 8;16(19):3246. doi: 10.3390/diagnostics16193246 (PMC13664159; doi:10.3390/diagnostics16193246)
Supplement: Supplementary file 1 [file diagnostics-16-03246-s001.zip › diagnostics-4528771-supplementary.pdf]

Supplementary File S1: Supplementary Analyses

Clinical Factors Associated with Diagnostic Delay in Axial Spondyloarthritis in Saudi Arabia: A Retrospective Cohort Study

Mohamed Bedaiwi, Aos Aboabat, Ibrahim Almaghlouth, Eman Alqurtas, Norah Alshafi

Cohort derivation: Of 220 registry records assessed for eligibility, 52 did not fulfill ASAS classification criteria and 8 had incomplete baseline registry data, leaving 160 eligible patients. All 160 had complete diagnostic-delay and primary-covariate data and were included in the present analyses without additional exclusions. The five primary covariates were selected before model fitting. The final analytic dataset was locked on 22 July 2026; no patients or data were added thereafter.

Supplementary Table S1. Characteristics by diagnostic-delay threshold

Characteristic	≤5 years (n=119)	>5 years (n=41)	p-value
Female sex	34 (28.6%)	22 (53.7%)	0.005
Age at symptom onset, years	29 (21–33)	33 (25–39)	0.009
Documented prediagnosis fibromyalgia	9 (7.6%)	15 (36.6%)	<0.001
Nonradiographic axSpA	49 (41.2%)	21 (51.2%)	0.279
HLA-B27 positive	76 (63.9%)	24 (58.5%)	0.578
Current smoking	25 (21.0%)	11 (26.8%)	0.516
BASDAI at index assessment	5.1 (4.3–5.9)	5.6 (5.2–6.9)	0.004
BASFI at index assessment	2.9 (2.2–3.7)	3.6 (2.6–4.5)	0.014
ASDAS-CRP at index assessment	3.5 (3.0–3.8)	3.6 (3.2–3.8)	0.056

Values are median (IQR) or n (%). Tests are descriptive and unadjusted. Disease-activity measures were recorded after diagnosis and should not be interpreted as consequences of delay.

Supplementary Table S2. Extended eight-variable negative binomial model

Predictor	Adjusted expected-delay ratio	95% CI	p-value
Female sex	1.03	0.82–1.30	0.776
Nonradiographic versus radiographic axSpA	1.13	0.91–1.41	0.250
HLA-B27 positive	1.01	0.81–1.24	0.957
Current smoking	0.93	0.73–1.18	0.563
Documented prediagnosis fibromyalgia	1.55	1.21–2.00	<0.001
Hypertension	1.29	0.99–1.67	0.059
BMI, per kg/m²	1.01	0.99–1.03	0.318
Age at symptom onset, per 5 years	1.07	1.00–1.14	0.066

This sensitivity model added current smoking, BMI, and hypertension measured at the later index assessment to the five primary variables. Expected-delay ratio denotes the exponentiated negative binomial coefficient, interpreted as the ratio of expected diagnostic delays.

Supplementary Table S3. Extended eight-variable logistic model for delay exceeding 5 years

Predictor	Adjusted OR	95% CI	p-value
Female sex	1.71	0.66–4.44	0.266
Nonradiographic versus radiographic axSpA	1.30	0.50–3.35	0.586
HLA-B27 positive	1.20	0.48–2.98	0.692
Current smoking	0.86	0.30–2.48	0.785
Documented prediagnosis fibromyalgia	5.42	1.97–14.93	0.001
Hypertension	1.72	0.58–5.04	0.325
BMI, per kg/m²	1.05	0.95–1.15	0.317

Age at symptom onset, per 5 years	1.26	0.94–1.68	0.121

Supplementary Table S4. Model diagnostics, bootstrap validation, and additional sensitivity checks

Analysis	Statistic	Estimate	Additional information
Primary negative binomial model	Poisson Pearson dispersion	1.75	–
Primary negative binomial model	Negative binomial alpha	0.121	p < 0.001
Primary negative binomial model	AIC	727.49	Poisson AIC 747.82
Primary model bootstrap	Converged resamples	1,995/2,000	Seed 20260725
Primary model bootstrap	Fibromyalgia expected-delay ratio	1.58	95% CI 1.21–2.08
Primary standardized predictions	No documented prediagnosis fibromyalgia	3.945 years	95% CI 3.52–4.37
Primary standardized predictions	Documented prediagnosis fibromyalgia	6.271 years	95% CI 4.84–8.06
Primary standardized contrast	Adjusted difference	2.33 years	95% CI 0.87–4.13
Interaction bootstrap	Converged resamples	1,997/2,000	Seed 20260725
Interaction bootstrap	Sex × fibromyalgia expected-delay ratio	1.94	95% CI 1.08–3.73
Firth penalized logistic sensitivity	Fibromyalgia OR	5.08	95% profile-likelihood CI 2.01–13.53; p < 0.001
Age functional-form sensitivity	3-knot restricted cubic spline	Likelihood-ratio p = 0.043	Knots: 18, 29, and 40 years; fibromyalgia expected-delay ratio 1.56 (95% CI 1.22–2.00)

Supplementary Table S5. Later index-assessment disease-activity measures by documented prediagnosis fibromyalgia status

Characteristic	No documented prediagnosis fibromyalgia (n=136)	Documented prediagnosis fibromyalgia (n=24)	p-value
BASDAI at index assessment	5.1 (4.3–5.7)	7.2 (6.9–7.8)	<0.001
BASFI at index assessment	2.9 (2.1–3.7)	4.0 (3.6–5.4)	<0.001
ASDAS-CRP at index assessment	3.5 (3.0–3.8)	3.9 (3.7–4.0)	<0.001

Values are median (IQR). These measures were recorded at the later index assessment and are presented descriptively; they are not interpreted as causes or consequences of the preceding diagnostic interval. Fibromyalgia status refers only to diagnoses documented before axSpA diagnosis; diagnoses after axSpA were not systematically ascertained. ASDAS-CRP, Ankylosing Spondylitis Disease Activity Score using C-reactive protein; BASDAI, Bath Ankylosing Spondylitis Disease Activity Index; BASFI, Bath Ankylosing Spondylitis Functional Index.

Supplementary Table S6. Exploratory sex-by-documented-prediagnosis-fibromyalgia analyses

Interaction estimates are from the five-variable negative binomial model plus the sex-by-fibromyalgia term. Standardized predictions retain the other covariates at observed values. Only eight men had documented prediagnosis fibromyalgia; all interaction findings are exploratory and hypothesis-generating. Fibromyalgia diagnosed after axSpA was not systematically ascertained.

Estimate	Expected-delay ratio or predicted delay	95% CI	p-value
Female sex × documented prediagnosis fibromyalgia	1.94	1.14–3.30	0.015
Prediagnosis fibromyalgia association among men	1.04	0.68–1.59	0.865
Prediagnosis fibromyalgia association among women	2.01	1.48–2.73	<0.001

Likelihood-ratio test for interaction	–	–	0.015
Bootstrap interaction estimate	1.94	1.08–3.73	–
Adjusted prediction: men without documented prediagnosis fibromyalgia	4.03 years	3.50–4.59	–
Adjusted prediction: men with documented prediagnosis fibromyalgia	4.18 years	2.52–6.30	–
Adjusted prediction: women without documented prediagnosis fibromyalgia	3.75 years	3.02–4.51	–
Adjusted prediction: women with documented prediagnosis fibromyalgia	7.54 years	5.63–9.87	–

Supplementary Table S7. Two-year landmark sensitivity analysis for fibromyalgia ascertainment

Item	Fibromyalgia documented by 2-year landmark	No fibromyalgia documented by 2-year landmark	95% CI	p-value
Landmark cohort, n	18	89	–	–
Median total diagnostic delay, years (IQR)	7.0 (5.3–8.0)	4.0 (3.0–6.0)	–	0.002
Median remaining delay after landmark, years (IQR)	5.0 (3.3–6.0)	2.0 (1.0–4.0)	–	0.002
Eventual diagnostic delay >5 years	13/18 (72.2%)	28/89 (31.5%)	–	0.003
Adjusted negative binomial association	Expected-delay ratio 1.51	Reference	1.05–2.19	0.027
Standardized expected remaining delay	4.88 years	3.22 years	–	–
Adjusted standardized difference	+1.66 years	Reference	0.23–3.74	–
Adjusted logistic association with delay >5 years	OR 4.66	Reference	1.35–16.04	0.015
Landmark bootstrap	1,999/2,000 converged	Seed 20260807	–	–

The landmark cohort was restricted to patients whose axSpA diagnosis occurred more than 2 years after symptom onset. Fibromyalgia was considered present at the landmark if first documented before symptom onset or within the first 24 months after symptom onset. The outcome for the negative binomial model was remaining diagnostic delay after the 2-year landmark. The adjusted models included sex, axSpA subtype, HLA-B27 status, landmark fibromyalgia status, and age at symptom onset. Among all 24 patients with documented prediagnosis fibromyalgia, 22 (91.7%) were documented by 2 years after axSpA symptom onset, including 3 (12.5%) documented before axial symptoms. Four of these 22 patients received an axSpA diagnosis within the first 2 years and were therefore ineligible for the landmark cohort, leaving 18 patients with fibromyalgia documented by the landmark; the remaining two prediagnosis fibromyalgia cases were first documented after 24 months and were classified as unexposed at the landmark. Descriptive continuous comparisons used the Mann–Whitney U test and proportions used Fisher's exact test.

Supplementary Figure S1. Adjusted diagnostic delay by sex and documented prediagnosis fibromyalgia status

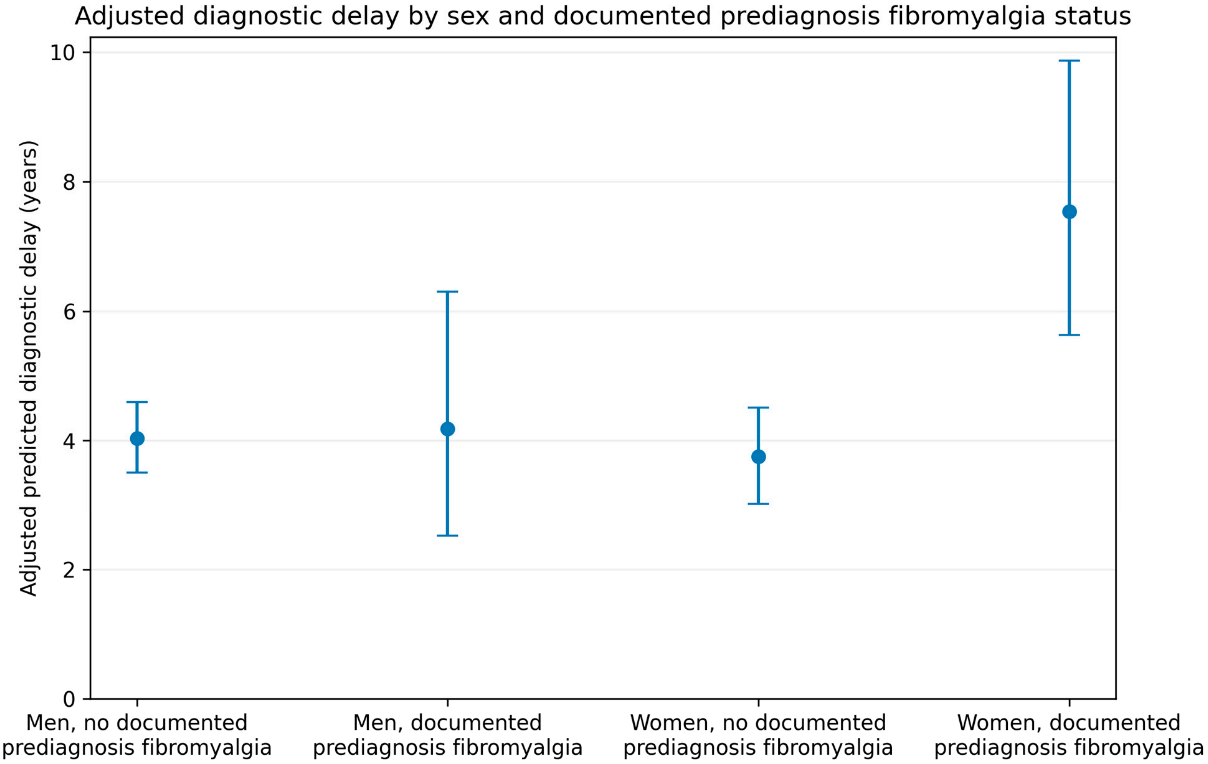

Points are standardized adjusted means from the exploratory five-variable interaction model; bars are percentile bootstrap 95% confidence intervals. The small subgroup of men with documented prediagnosis fibromyalgia limits precision. Postdiagnosis fibromyalgia was not systematically ascertained.

Supplementary Figure S2. Adjusted relationship between age at axSpA symptom onset and predicted diagnostic delay

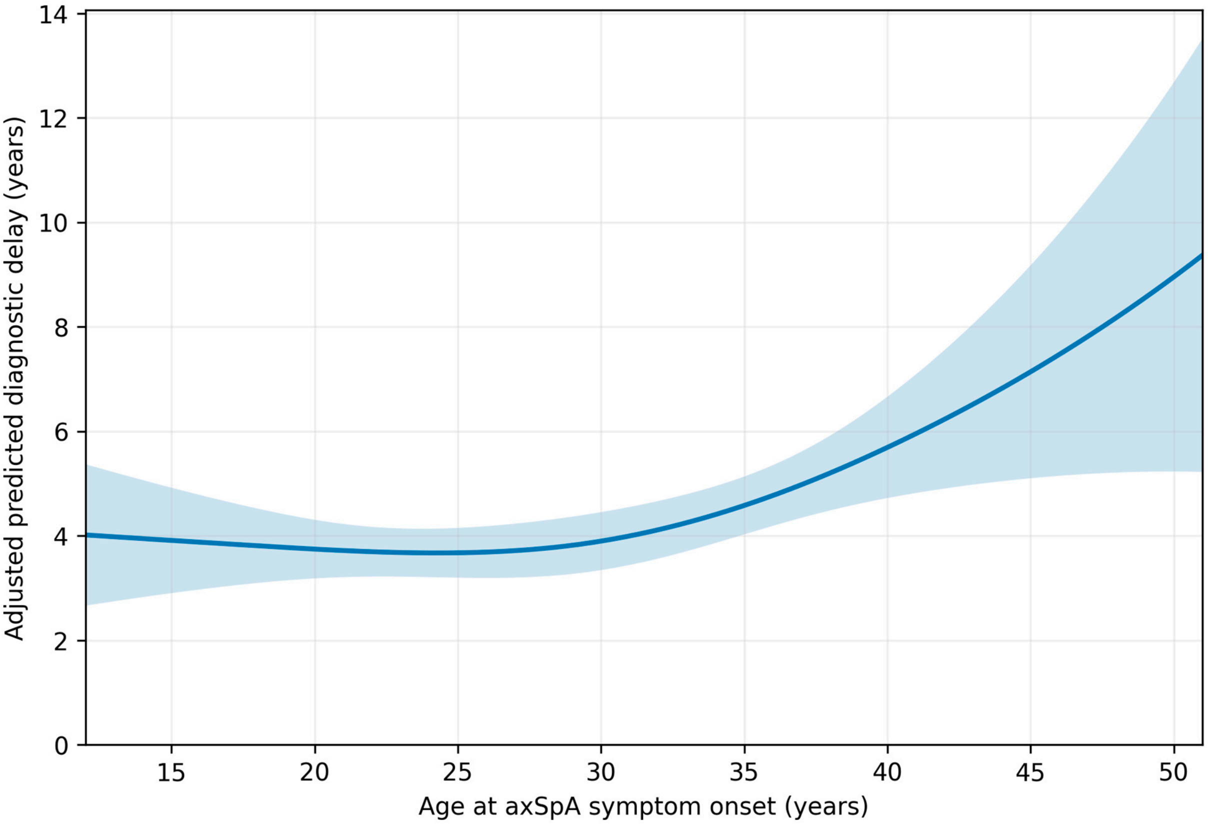

Adjusted predicted diagnostic delay was estimated using a negative binomial sensitivity model incorporating symptom-onset age as a three-knot restricted cubic spline, with knots at the 10th, 50th, and 90th percentiles (18, 29, and 40 years). Predictions were standardized over the observed distributions of sex, axSpA subtype, HLA-B27 status, and documented prediagnosis fibromyalgia. The solid line represents adjusted predicted diagnostic delay and the shaded area the 95% confidence interval. Evidence of departure from strict log-linearity was modest (likelihood-ratio p = 0.043).
